# A Japan-origin motivational framework for diversive and specific curiosity: development of the English version of the Japanese Epistemic Curiosity scale

**DOI:** 10.3389/fpsyg.2026.1762069

**Published:** 2026-04-20

**Authors:** Kazuji Nishikawa, Ryota Kanai, Hiro Taiyo Hamada, Jamie Ward, Takashi Kusumi

**Affiliations:** 1 Faculty of Business Administration, Department of Commerce, Osaka University of Commerce, Osaka, Japan; 2 Kyoto University, Institute for Liberal Arts and Sciences, Kyoto, Japan; 3 Araya Inc., Tokyo, Japan; 4 School of Psychology, University of Sussex, Brighton, United Kingdom

**Keywords:** curiosity, diversive curiosity, epistemic curiosity, exploration, openness to experience, specific curiosity

## Abstract

Curiosity is a fundamental human drive to explore, the desire to know. In particular, epistemic curiosity is the central construct in individual differences in curiosity. Epistemic curiosity represents a manifestation of exploratory behavior within the intellectual and cognitive domains. Japanese studies in epistemic curiosity have developed independently, based on Hatano and Inagaki’s theory. Studies have been conducted on the role of learning and education for the two types of epistemic curiosity, diversive and specific curiosity. The Japan version of the Epistemic Curiosity (J-EC) scale, which measures these two types as a personality trait, has been developed based upon empirical studies using the J-EC scale. We thought it was necessary to develop an English version of the J-EC scale, in order to be able to make international comparisons of levels of curiosity among Japanese people. In this study, we translated the J-EC scale into English and assessed its factor structure, reliability, and validity. Results of factor analysis and correlation analysis validated the factor structure of diversive and specific curiosity, and the reliability and validity of diversive and specific curiosity subscales almost perfectly in accordance with our hypotheses.

## 1 Introduction

Curiosity has long been recognized as a basic drive common to mammals (Berlyne, 1960; Loewenstein, 1994; Panksepp, 1998). As such, curiosity pertains to several areas of human activity from daily life to scientific creativity. Curiosity has been shown to enhance memory (Kang et al., 2009) and has been associated with higher academic achievement (Mussel, 2022) and job performance (Mussel, 2013). It also plays a role in fostering interpersonal relationships (Kashdan and Roberts, 2006), and improving subjective well-being (Kashdan et al., 2020). These findings are supported by a substantial body of research focused on the measurement of curiosity. In particular, curiosity has been conceptualized as a multidimensional construct, contributing to our understanding of individual differences. Among these dimensions, epistemic curiosity—defined as the desire to acquire new knowledge and resolve uncertainty—has emerged as the most important dimension (Mussel, 2013).

The present study aimed to validate the English version of the Japanese Epistemic Curiosity Scale (Nishikawa and Amemiya, 2023, 2018), which distinguishes between two motivational types of curiosity: diversive and specific. While the J-EC scale has demonstrated reliability and utility in educational research in Japan, its applicability in English-speaking contexts has not yet been examined. By investigating its factor structure, reliability, and convergent validity, this study establishes the J-EC scale as a cross-culturally valid instrument for assessing epistemic curiosity, thereby contributing to the generalizability of the epistemic curiosity scale.

In the present study, we focus specifically on epistemic curiosity as a motivational disposition toward knowledge acquisition, rather than perceptual curiosity or affective curiosity constructs.

### 1.1 Theoretical background

#### 1.1.1 Classical theories of curiosity

Although curiosity has been acknowledged since ancient times (James, 1890; McDougall, 1908), experimental psychological research on curiosity began in the 1950s with the work of Berlyne. Berlyne (1960) defined curiosity using behaviorist principles. Specifically, he claimed that person-specific curiosity, such as the “drive to know,” involving intellectual activities such as thinking and ideas, is distinct from curiosity shared by humans and animals, triggered by excitement or stimulation. The former is termed “epistemic curiosity” and the latter, “perceptual curiosity.” Berlyne also conceptualized curiosity as the motivation underlying exploratory behavior, which is aroused by collative variables such as novelty, complexity, uncertainty, and conflict. He further identified two types of exploratory behavior, diversive exploration, which arises from boredom and seeks novelty, and specific exploration, which aims to resolve uncertainty or conflict. Berlyne (1971) regarded curiosity as a characteristic of specific exploration, but did not address its relationship with diversive exploration. Loewenstein (1994) expanded upon Berlyne’s ideas with “the information gap theory,” suggesting that curiosity arises from the discomfort caused by a gap between what one knows and what one wants to know. Resolving this gap brings pleasure, even though the state of curiosity itself may feel uncomfortable. Panksepp (1998) proposed that the SEEKING system, one of six emotional brain systems shared by humans and mammals, represents the biological foundation of human curiosity. This system drives exploratory behavior through instinctive interest and excitement when anticipating desired outcomes, forming the basis for seeking everything from food and relationships to scientific ideas. Beswick (1971, 2017) focused on cognitive processes underlying curiosity, drawing particularly influence from Piaget’s concepts of assimilation and accommodation. He emphasized that the learning process involves both adapting to novel stimuli and repeatedly engaging acquired information until it is fully understood. From these two processes, he proposed two cognitive strategies of curiosity: “openness (to novelty)” and “interest in orderliness.” Spielberger and Starr (1994) explored the relationship between two types (diversive/specific) of curiosity and anxiety, proposing that curiosity leads to exploration when it outweighs anxiety, and vice versa. Other researchers introduced the concepts of breadth (wide-ranging interest) and depth (focused investigation) in curiosity-driven exploration (Langevin, 1971; Ainley, 1987).

#### 1.1.2 Challenges in measuring epistemic curiosity

Although curiosity has been conceptualized as a multidimensional construct in research on individual differences (Grossnickle, 2014), epistemic curiosity is widely recognized as its central and most influential dimension (Mussel, 2013). The Epistemic Curiosity Scale (ECS; Litman and Spielberger, 2003), based on Berlyne’s framework, includes diversive and specific subscales. However, the validity of the ECS has been questioned, as many studies treat it as a unified construct and report similar correlations with external measures such as trait anxiety and openness (Mussel, 2010). The ECS has also been used to examine the relationship between trait anxiety and the two types of curiosity. Spielberger and Starr (1994) noted the coexistence of curiosity and anxiety, with specific curiosity being more positively associated with anxiety than diversive curiosity ([correlation with the specific curiosity scale] > [correlation with the diversive curiosity scale]). However, a study using the ECS reported exactly the same correlation (−0.15) with trait anxiety for both diversive and specific curiosity (Litman and Spielberger, 2003). These findings are inconsistent with the theoretical predictions discussed above. Contemporary research on epistemic curiosity has been modernly revived and operationalized through a new Epistemic Curiosity Scale (Litman, 2008). This development primarily centers on the distinction between Interest-type (I-type) and Deprivation-type (D-type) curiosity. This theoretical framework is grounded in Information-Gap theory (Loewenstein, 1994) and the conceptualization of Curiosity as a Feeling of Interest (CFI)—as observed in typical trait curiosity scales that measure curiosity as affective states (Litman and Jimerson, 2004), such as the Values In Action (VIA; Peterson and Seligman, 2001). It conceptualizes curiosity in affective terms, distinguishing intrinsic enjoyment (such as I-type) from the aversive experience of information deprivation (such as D-type): a “gap” in knowledge that requires resolution.

While this affect-based distinction has catalyzed extensive research, recent theoretical discussions have increasingly emphasized process-oriented or regulatory interpretations of curiosity rather than a unitary affective state (McNary, 2023). This perspective focuses on the optimization of cognitive engagement while acknowledging “non-curiosity” states, such as avoidance triggered by informational overload or excessive uncertainty. Such contemporary trends align closely with the regulatory framework pioneered by Hatano and Inagaki (1971) —the details of which are discussed in the following section— which emphasizes the regulation of information-processing levels based on conceptual conflict. Their model thus anticipated modern efforts to resolve measurement issues by highlighting the internal mechanisms of intellectual engagement.

### 1.2 Curiosity research in Japan

#### 1.2.1 Hatano and Inagaki’s theory

Based on the regulatory perspective discussed earlier, Hatano and Inagaki (1971) developed a distinctive theoretical framework that reframes epistemic curiosity as a mechanism for the internal regulation of information processing. While their work initially drew on Berlyne’s (1960) foundational theory, they significantly expanded it by focusing on the functional roles of diversive and specific curiosity in optimizing cognitive engagement. Unlike affect-centered models, this framework emphasizes how these two types of curiosity serve as intellectual strategies to manage the level of informational stimulation and resolve conceptual conflict, particularly within educational contexts. Hatano and Inagaki (1971) highlighted differences in the motivational mechanisms underlying the two types of epistemic curiosity and articulated these distinctions in their framework. They proposed two hypotheses regarding the types of curiosity, based on motivational systems observed in humans and higher animals. Hatano and Inagaki (1971), drawing on experiments by Heron (1957) and Harlow (1950), which demonstrated exploratory behavior independent of external rewards, argued that human exploratory behavior —whether seeking information about novel stimuli or attempting to solve unsolvable puzzles—is fundamentally characterized by active engagement with novelty. They proposed that humans and higher animals are inherently curious beings and posited the existence of a foundational motivational system rooted in intrinsic curiosity. The theoretical framework supporting this foundational system, along with mechanisms underlying both diversive and specific curiosity, was called “cognitive motivation.”

**Figure 1:**
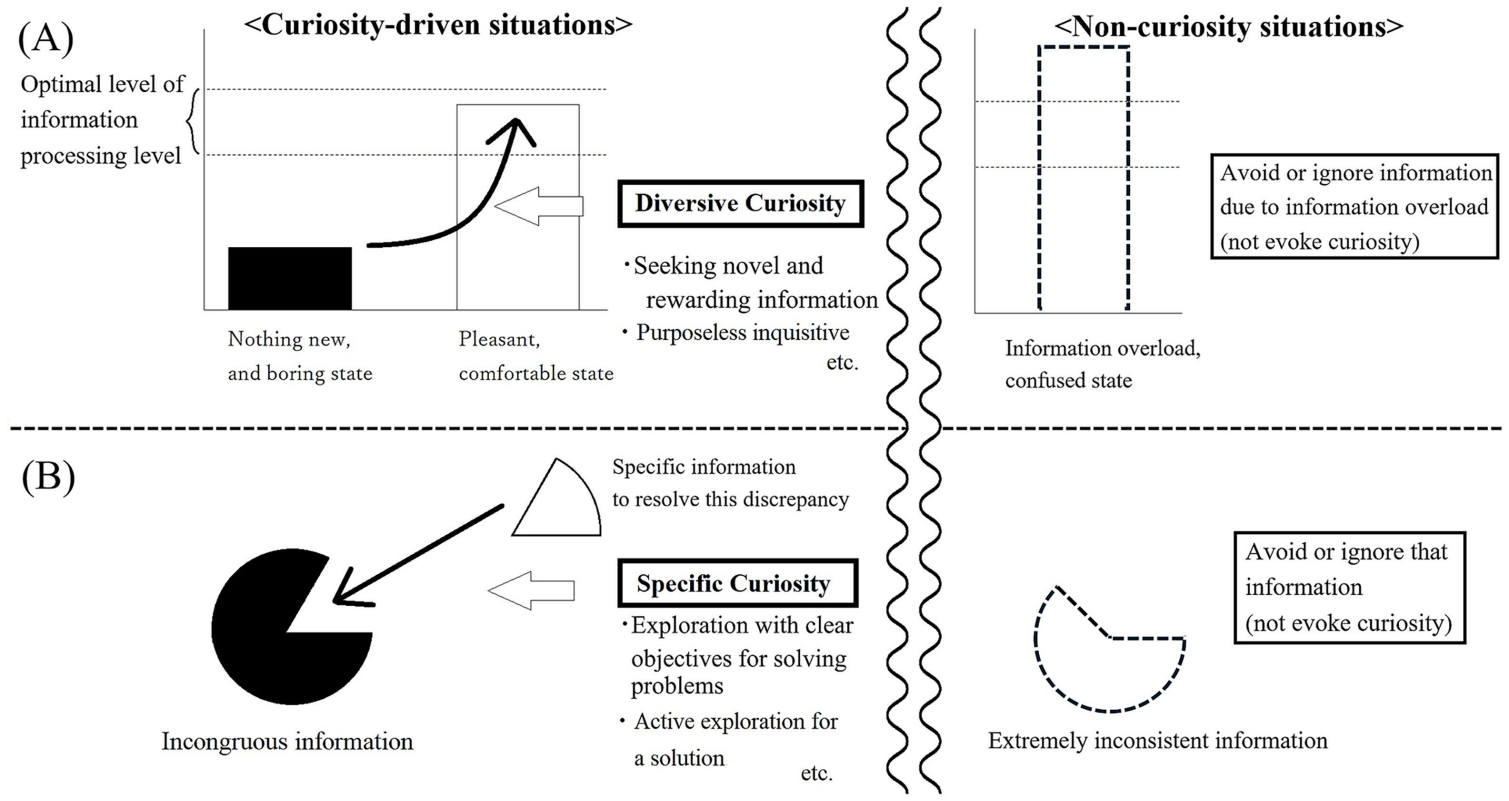
Graphic explanation of motivational mechanisms of diversive and specific curiosity, according to the theory of Hatano and Inagaki. **(A)** The upper panel illustrates diversive curiosity, which is triggered when the level of information processing falls below the optimal threshold, resulting in boredom due to a situation lacking novelty. This motivates individuals to seek novel and rewarding experiences through purposeless inquisitive exploration (diversive curiosity-driven situation). However, when the amount of information exceeds the optimal level (i.e., information overload, depending on individual differences), it is unlikely that individuals will be motivated to restore the optimal state. Instead, they tend to avoid or ignore such excessive information (non-curiosity situation). **(B)** The lower panel depicts specific curiosity, which arises when individuals encounter incongruous or incomplete information. This state motivates focused exploration aimed at resolving uncertainty or finding solutions to specific problems (specific curiosity-driven situation). However, if the degree of information discrepancy is too large (depending on individual differences), curiosity to resolve the inconsistency may not arise, and individuals may instead avoid or ignore such information (non-curiosity situation). Both mechanisms are conceptualized as distinct but complementary motivational drives underlying epistemic curiosity.

First, individuals possess a certain level of information-processing drive, which depends on their abilities, past experiences (such as preferences), and the current situation. When the amount of incoming information falls below the optimal level for information processing, individuals become bored and initiate diversive exploration to increase stimulation to an optimal level—this is diversive curiosity. This motivational mechanism is derived from McReynolds (1962) theory of the “optimal level of perceptualization rate.” Hatano and Inagaki (1971) replaced “perceptualization” with the broader term “information processing” and proposed an optimal-level theory of information processing. The tendency of diversive curiosity-driven exploration includes seeking novel and enjoyable information, engaging in purposeless inquiry, and creating new information by interacting with the environment or altering one’s perspective. In addition, exceeding the optimal level of incoming information encourages avoidance behavior. Second, individuals possess a cognitive drive based on the desire to resolve incongruities by seeking desired information or reformulating the current problem. When faced with uncertainty, individuals are motivated to engage in specific exploration aimed at resolving these discrepancies—this is specific curiosity. Hatano and Inagaki (1971) built upon Berlyne’s conceptual conflict model but emphasized that specific curiosity does not necessarily arise from unpleasant feelings. Instead, it is triggered by cognitive incongruity, such as uncertainty or inconsistency, without emotional discomfort. The primary triggers for this exploration are focused on incongruous situations. The tendency to specific curiosity exploration includes exploration with clear objectives for problem-solving, active exploration for a solution, and a high degree of persistence in the exploratory behavior. These basic mechanisms are illustrated in Figure 1.

Beyond its historical origin, Hatano and Inagaki’s framework offers several theoretically distinctive contributions. First, it characterizes curiosity as involving a regulatory function for managing information-processing levels, rather than primarily as affective arousal or hedonic experience—a perspective that remains highly relevant to contemporary theoretical discussions that question affect-based and unitary conceptions of curiosity (e.g., McNary, 2023). Diversive curiosity is conceptualized as restoring an optimal level of informational stimulation, whereas specific curiosity emerges from cognitive discrepancy requiring resolution. Second, the model explicitly incorporates non-curiosity states, such as avoidance triggered by informational overload or extreme inconsistency—conditions under which exploration is suppressed rather than activated. Third, diversive and specific curiosity are not positioned along a single affective continuum but are modeled as complementary motivational systems that jointly shape intellectual engagement. This regulatory framework provides a structurally distinct alternative to more traditional, affect-based dual-process models of epistemic curiosity.

#### 1.2.2 Development of the J-EC scale

The Japanese Epistemic Curiosity (J-EC) scale was developed to operationalize this regulatory framework. Nishikawa and Amemiya (2015) developed the original Japanese Epistemic Curiosity (J-EC) scale to measure both diversive and specific curiosity, drawing on the theoretical framework of Hatano and Inagaki (1971). Previous studies had developed the Epistemic Curiosity Scale (ECS; Litman and Spielberger, 2003), which includes two subscales—diversive and specific curiosity—focused on the breadth and depth of exploratory behavior. However, the validity of these subscales has been questioned (Mussel, 2010).

In contrast, the J-EC scale explicitly distinguishes between diversive and specific curiosity (Hatano and Inagaki, 1971). It focuses on two aspects of cognitive motivational processing: seeking novelty and resolving information discrepancies. The J-EC scale (Nishikawa and Amemiya, 2015) consists of diversive and specific curiosity subscales, reflecting novelty-seeking and discrepancy-resolution tendencies. Validation studies confirmed its two-factor structure and revealed distinct patterns of correlation. Diversive curiosity scale was strongly associated with BAS (Behavioral Activation System) Fun-Seeking scale (0.52), whereas specific curiosity scale was correlated with NFC (Need For Closure) Preference for Order scale (0.31), indicating clear functional differences.

Recent research in Japan has further broadened the scope of epistemic curiosity studies. For example, Nishikawa and Amemiya (2017) demonstrated that both diversive and specific curiosity traits influence the appraisal of picture stimuli, revealing subtle effects of curiosity on emotional responses. More recently, Nishikawa and Amemiya (2023) reported that these two types of epistemic curiosity exert distinct influences on aesthetic experiences, offering new insights into how diversive curiosity and specific curiosity contribute to engagement with art and design.

#### 1.2.3 Broader applications of epistemic curiosity research

In Japan, a growing body of empirical research has utilized the J-EC scale to investigate curiosity and its role in educational settings. For example, students with a high level of specific curiosity tend to maintain greater interest in classroom activities (Okibayashi, 2018). Students with high levels of diversive curiosity subscale had slightly more days for English e-learning (Abe and Kogo, 2021). Moreover, education emphasizing critical thinking in elementary and junior high school classrooms leads to an increase in the J-EC (Unzai et al., 2020). In addition, Totsune et al. (2021) demonstrated that diversive curiosity contributes to enhanced subjective well-being, while Sekiguchi (2023) found that diversive curiosity positively predicts both intentional and unintentional mind wandering. These findings clearly illustrate the distinct functional roles of the two types of epistemic curiosity, as identified through the application of the J-EC scale. The J-EC scale has addressed previous measurement challenges related to diversive and specific curiosity, and it has been widely adopted in educational research and public organizations in Japan (Nishikawa and Kusumi, 2023; Nishikawa et al., 2023).

### 1.3 Purpose of the present study

Previous research on curiosity has largely been conducted independently in Japan. We considered it valuable to validate the reliability and validity of the J-EC scale in English-speaking regions. Therefore, the present study aimed to validate the English version of the Japanese Epistemic Curiosity (J-EC) scale by examining its factor structure, reliability, and convergent validity. This cross-cultural validation will contribute to the generalizability of curiosity research and provide a reliable tool for assessing epistemic curiosity in English-speaking populations. We proposed the following hypotheses:

#### 1.3.1 Hypothesis A

The English version of the Japanese Epistemic Curiosity (J-EC) scale exhibits a two-factor structure, consistent with the original Japanese version. These two factors correspond to diversive curiosity and specific curiosity. It is further expected that both subscales will demonstrate high internal consistency, with Cronbach’s alpha coefficients exceeding 0.70.

#### 1.3.2 Hypothesis B

The English version of the J-EC scale demonstrates convergent validity through significant positive correlations with theoretically related constructs. Specifically, both subscales are hypothesized to be positively correlated with the Openness to Experience dimension of the Big Five personality traits, the Need for Cognition Scale (NCS), and Curiosity and Exploration Inventory-II (CEI-II), as both have been conceptualized, relative to curiosity (Kashdan, 2004). Additionally, a positive association is anticipated with the Behavioral Activation System (BAS) Drive scale, which reflects goal-directed approach behavior and is conceptually aligned with exploratory tendencies inherent in curiosity (Carver and White, 1994).

#### 1.3.3 Hypothesis C

The diversive curiosity subscale is positively associated with the BAS Fun-Seeking scale. This subscale captures a tendency to seek novel and stimulating experiences (Nishikawa and Amemiya, 2015), which aligns with the BAS Fun-Seeking dimension that reflects a proclivity for spontaneous pursuit of potentially rewarding experiences (Carver and White, 1994).

#### 1.3.4 Hypothesis D

The specific curiosity subscale is positively associated with the Preference for Order scale of the Need For Closure scale. Specific curiosity reflects a desire to resolve uncertainty and ambiguity information, and to acquire precise information, which is conceptually related to a preference for structure and order in cognitive processing.

## 2 Methods

### 2.1 Participants

A total of 687 undergraduate students (*M* age = 19.73 years, *SD* = 2.30; 572 women, 109 men, and six others) from the University of Sussex, UK, participated in this study. All participants were 18 years or older.

### 2.2 Procedure and data collection

Participants were recruited via the School of Psychology’s online participation panel and completed the study through a web-based questionnaire platform (SurveyMonkey). The determination of sample size in SEM (Structural Equation Modeling) should consider the stability of model fit indices. According to Shi et al. (2019), a sample size of less than 500 may lead to unstable estimates. Data collection was stopped once the predetermined target sample size of approximately 600 participants was reached, based on practical constraints and recommendations for stable SEM estimation. In the verification of correlation analysis, effect sizes are determined based on Cohen (1988): small (*r* = 0.10–0.29), medium (*r* = 0.30–0.49), and large (*r* ≥ 0.50).

### 2.3 Materials and procedure

#### 2.3.1 Japanese Epistemic Curiosity (J-EC) scale

The J-EC (Nishikawa and Amemiya, 2015, 2018) scale was developed as an original Japanese epistemic curiosity scale to measure diversive curiosity and specific curiosity. J-EC scale contains two 6-item subscales: the diversive curiosity subscale and the specific curiosity subscale. Items are rated on a 5-point scale, ranging from 1 (not at all) to 5 (extremely). Both subscales have sufficient reliability (α = 0.81 for both subscales) and adequate validity in relation to other basic measures, e.g., Big Five scale- Openness to Experience subscale, NCS (Need for Cognition Scale), NFC (Need For Closure) scale - Preference for Order subscale, and subscales of the BIS/BAS (Behavioral Inhibition System/Behavioral Activation System). An English version of the J-EC scale was back-translated by a native English speaker.

#### 2.3.2 20-item Mini-International Personality Item Pool (Mini-IPIP)

The Mini-IPIP (Donnellan et al., 2006) contains five 5-item subscales: Openness to Experience, Conscientiousness, Extraversion, Agreeableness, and Neuroticism. Items are rated on a 5-point scale, ranging from 1 (very inaccurate) to 5 (very accurate). The correlation analysis in the present study was conducted using only the Openness to Experience scale, which is relevant to Hypothesis B.

#### 2.3.3 Curiosity and Exploration Inventory-II (CEI-II)

The CEI-II (Kashdan et al., 2009) assesses individual differences in the tendency to experience curiosity and to seek out novel experiences. This scale consists of 10 items. Items are rated on a 5-point scale, ranging from 1 (very slightly or not at all) to 5 (extremely). In the present study, we conducted a correlation analysis using the total CEI-II score, which is relevant to Hypothesis B.

#### 2.3.4 Need for Cognition Scale (NCS)

The NCS (Cacioppo and Petty, 1982) was developed to measure a tendency to engage in and enjoy cognitive activities, such as abstract thinking, deep contemplation, and problem-solving. This scale consists of 17 items. Items are rated on a 7-point scale, ranging from 0 (strongly disagree) to 6 (strongly agree). The correlation analysis in the present study was conducted using the NCS, which is relevant to Hypothesis B.

#### 2.3.5 15-item version of the Need For Closure scale (15 items-NFC scale)

The 15 item-NFC (Roets and Van Hiel, 2011) scale is a brief version of the Need For Closure scale (Webster and Kruglanski, 1994). The NFC contains four subscales: Preference for Order, Predictability, Decisiveness, and Ambiguity, Closed-mindedness. Items are rated on a 5-point scale ranging from 1 (completely disagree) to 5 (completely agree). The correlation analysis in the present study was conducted using only the Preference for Order scale, which is relevant to Hypothesis D.

#### 2.3.6 Behavioral Inhibition System/Behavioral Activation System scale (BIS/BAS scale)

The BIS/BAS (Carver and White, 1994) scale was developed to measure a tendency toward Behavioral Inhibition System (BIS), which avoidance motivates, and tendency toward Behavioral Activation System (BAS), which is an appetitive motive and approach. The BAS contains three subscales: BAS Drive, BAS Fun-Seeking, and BAS Reward. This scale consists of 20 items. Items are rated on a 4-point rating scale, ranging from 1 (very false for me) to 4 (very true for me). The correlation analysis in the present study was conducted using only the BAS Drive and BAS Fun-Seeking, which is relevant to Hypothesis B and C.

## 3 Results

### 3.1 Factor analysis and reliability

Prior to conducting the Exploratory Factor Analysis (EFA), the suitability of the data for factor analysis was assessed. The Kaiser-Meyer-Olkin (KMO) measure of sampling adequacy was 0.849, which is well above the recommended threshold of 0.60, and Bartlett’s test of sphericity was statistically significant [*χ*^2^(66) = 1849.93, *p* < 0.001]. These results indicate that the correlation matrix was factorable and that the data were appropriate for EFA.

First, to examine the factor structure of the English version of the J-EC, we conducted an EFA using maximum likelihood estimation with promax rotation. Each eigenvalue was as follows (from the 1st to the 6th): 3.86, 1.59, 0.96, 0.84, 0.77, and 0.70. Based on the scree plot criterion, a two-factor solution was retained, explaining 35.0% of the total variance. Using a loading criterion of 0.40 or higher, six items loaded onto the specific curiosity factor and six items loaded onto the diversive curiosity factor (Table 1). There were no items that showed cross-loadings of 0.30 or higher on the two factors.

**Table 1:** Exploratory factor analysis of 12 items in the Japanese Epistemic Curiosity Scale (Maximum-likelihood method, Promax rotation, *N* = 687).

Itm No.		Factor				
	Items	SC	DC	*h* ^*2*^	*M*	*SD*
11.	I will think many hours to solve a problem.	**0.68**	-0.04	0.43	3.13	1.15
3.	I will never be satisfied until I have acquired all necessary knowledge about an idea.	**0.60**	-0.02	0.35	3.33	1.07
6.	I will think through a problem, till a clear and definite answer emerges.	**0.60**	-0.08	0.32	3.66	0.93
4.	When I can’t reach the solution, I am uneasy and eager to reach it.	**0.59**	-0.10	0.30	3.87	0.88
12.	When I learn things, I want to inquire thoroughly.	**0.48**	0.16	0.34	3.63	0.93
8.	When something unexpected has happened, I will check until I find out the cause.	**0.40**	0.09	0.21	3.59	0.95
5.	Wherever I go, I explore new things and new experiences.	-0.18	**0.70**	0.39	3.72	0.94
1.	I explore new ideas in various ways.	-0.03	**0.70**	0.47	3.53	0.93
2.	I like to challenge myself with new things.	-0.07	**0.69**	0.43	3.55	1.00
9.	I am very curious about the things that nobody has tried yet.	0.23	**0.43**	0.34	3.23	1.10
7.	I am curious about everything.	0.16	**0.42**	0.27	3.79	0.98
10.	I am willing to work on a task that nobody has tried yet.	0.27	**0.40**	0.34	3.24	1.02
				Scale analysis		
		SC	DC	*α*	*M*	*SD*
	SC	―	0.52^a^	0.73	3.54	0.65
	DC	0.45^b^	―	0.76	3.51	0.67

SC, specific curiosity; DC, diversive curiosity; h^2^, communality; a inter-factor correlation; b subscale correlation; *α*, Cronbach’s alpha coefficients.

To further evaluate the factor structure and establish factorial validity, a Confirmatory Factor Analysis (CFA) was conducted. The initially specified two-factor model demonstrated acceptable but slightly suboptimal fit, *χ*^2^ (53) = 257.949, *p* < 0.001; CFI = 0.886, GFI = 0.937, NFI = 0.862, RMSEA = 0.075 (90% CI: [0.066, 0.084]). This model was compared with a single-factor model, which exhibited poor fit, *χ*^2^ (54) = 491.34, *p* < 0.001; CFI = 0.757, GFI = 0.868, NFI = 0.736, RMSEA = 0.109 (90% CI: [0.100, 0.118]), indicating that the two-factor structure provided a substantially better representation of the data. Inspection of modification indices suggested correlated residuals among the three items within the diversive curiosity factor (item-1–item-2, item-1–item-5, item-2–item-5). Allowing these within-factor residual covariances suggested by the modification indices improved model fit considerably, *χ*^2^ (50) = 138.48, *p* < 0.001; CFI = 0.951, GFI = 0.968, NFI = 0.926, RMSEA = 0.051 (90% CI: [0.041, 0.061]). Importantly, the overall two-factor structure and pattern of factor loadings remained unchanged. Therefore, the original theoretically specified two-factor model was retained as the primary model, and the modified model is reported as supplementary evidence of structural robustness.

Regarding reliability, Cronbach’s alpha coefficients were 0.76 for the diversive curiosity subscale and 0.73 for the specific curiosity subscale. In addition, McDonald’s omega coefficients, (calculated using standardized factor loadings from the two-factor CFA model without correlated residuals), were 0.76 for diversive curiosity and 0.74 for specific curiosity. These results indicate acceptable internal consistency for both subscales and support the reliability of the English version of the J-EC scale.

### 3.2 Correlation analysis

Results of the correlation analysis (Table 2) were confirmed from hypotheses B to D. We treated the correlations specified *a priori* in Hypotheses B–D as confirmatory tests and applied a Bonferroni correction within each hypothesis-specific set of comparisons (*α* = 0.05/12 = 0.004). The results were substantively unchanged, with all primary correlations remaining statistically significant after correction. Both subscales (Diversive /Specific) of the English version of the J-EC scale correlated positively with the Openness scale, the CEI- II, the Need for Cognition Scale, and BAS Drive scale: these results supported hypothesis B. The diversive curiosity subscale was positively correlated with the BAS Fun-Seeking scale: this result supported hypothesis C. The specific curiosity subscale was positively correlated with the Preference for Order scale: this result supported hypothesis D.

**Table 2:** Correlation between the openness to experience, the need for cognition, the preference for order, bis/bas and the japanese epistemic curiosity scale (*N* = 687).

	Diversive Curiosity	Specific Curiosity
Scales	*r* [95%CI]	*r* [95% CI]
Big Five Scale		
Openness to Experience	0.34 [0.28, 0.41]^***^	0.25 [0.18, 0.32]^***^
Curiosity scale		
Curiosity and Exploration Inventory-II (CEI-II)	0.68 [0.63, 0.71]^***^	0.28 [0.21, 0.35]^***^
Need for Cognition Scale (NCS)		
Need for Cognition	0.52 [0.47, 0.58]^***^	0.54 [0.48, 0.59]^***^
Need For Closure (NFC) scale		
Preference for Order	–0.15 [–0.22, –0.07]^***^	0.18 [0.10, 0.25]^***^
Behavioral Inhibition System/Behavioral Activation System scale (BIS/BAS scale)		
BAS Drive	0.38 [0.31, 0.44]^***^	0.28 [0.21, 0.35]^***^
BAS Fun-Seeking	0.42 [0.35, 0.48]^***^	–0.05 [–0.12, 0.03]

*r*, Pearson’s correlation coefficient; CI, Confidence Interval.

*** *p*<.001.

## 4 Discussion

The present validation therefore not only extends the J-EC to an English-speaking context but also underscores the theoretical relevance of Hatano and Inagaki’s regulatory model of epistemic curiosity. The purpose of this study was to develop an English version of the J-EC scale. To this end, we formulated four hypotheses based on hypotheses developed for the J-EC scale, and confirmed them.

The positive correlation (0.18) between the specific curiosity subscale and the Preference for Order scale was not as high as the correlation (0.31) in Japan (Nishikawa and Amemiya, 2015). Meanwhile, the correlation between the diversive curiosity scale and the preference for order scale did not show a positive correlation. We found that specific curiosity was more likely to seek out well-organized information than diversive curiosity. This finding reinforces the conceptualization of specific curiosity as a type of epistemic curiosity that seeks to resolve information inconsistencies. In the J-EC scale, specific curiosity and diversive curiosity are conceptualized as dual motivational drives, analogous to the two drive wheels of a vehicle, that propel human intellectual activity (Hatano and Inagaki, 1971; Nishikawa and Amemiya, 2015, 2018). This dual-structure aligns with Beswick’s (1971, 2017) cognitive framework of curiosity, particularly the dimensions of “(openness to) novelty” and “(interest in) orderliness.” The nature of specific curiosity, characterized by a drive to resolve inconsistencies and contradictions, has been central to exploring human problem-solving processes. However, such processes pre-suppose a foundation of accumulated knowledge and prior information. In this context, diversive curiosity, which reflects a broad exploratory tendency toward novel stimuli and information, plays a complementary and essential role (Arnone et al., 2011). Thus, it requires consideration of both specific and diversive forms of curiosity. The J-EC scale is a valuable tool for understanding how multidimensional aspects of curiosity contribute to cognitive engagement.

### 4.1 Limitations

The findings of this study provide preliminary evidence supporting the proposed framework (Figure 1). Furthermore, the utility and effectiveness of the scale—particularly its ability to distinguish between the two types of curiosity—require further investigation. One limitation is that the model fit indices and total variance explained (35.0%) were not fully conclusive. However, these results are consistent with previous Japanese research (Nishikawa and Amemiya, 2015) and the factor structure remained highly distinct with no significant cross-loadings. This suggests that the primary latent constructs were effectively captured, supporting the scale’s practical applicability. Future research should aim to improve these indices by further refining the English item expressions to better capture the nuances of the original Japanese constructs. This discrepancy may stem from issues related to item translation or cultural differences between Japanese and English-speaking samples. Future studies should focus on refining the English version and addressing challenges in translation and cultural adaptation. To further demonstrate the utility and effectiveness of this scale, it will be necessary to empirically examine cognitive and educational differences between diversive and specific curiosity—using experimental and educational surveys as previously conducted in Japanese studies.

### 4.2 Conclusion

Despite these limitations, the present study is the first to validate the Japanese Epistemic Curiosity (J-EC) scale in English, providing a valuable tool for assessing individual differences in curiosity in cross-cultural research. The English version of the J-EC scale enables direct comparison of curiosity between Japanese and English-speaking participants, thereby deepening our understanding of the role of curiosity across different cultural backgrounds. By elucidating the universal mechanisms of diversive and specific curiosity, this study offers a new perspective to traditional research on epistemic curiosity. This advancement not only broadens the practical applications of curiosity research in educational settings, but also contributes to our understanding and support of human intellectual exploration and information-seeking behavior. Furthermore, the findings offer new insights for future research in related fields such as gaming (Pathak et al., 2017; Tang and Kirman, 2025) and robotics (Gordon, 2020).

## Data Availability

The raw data supporting the conclusions of this article will be made available by the authors, without undue reservation.
