# Supplementary material for: A Japan-origin motivational framework for diversive and specific curiosity: development of the English version of the Japanese Epistemic Curiosity scale
Source: Front Psychol. 2026 Apr 20;17:1762069. doi: 10.3389/fpsyg.2026.1762069 (PMC13136233; doi:10.3389/fpsyg.2026.1762069)
Supplement: Supplementary file 1 [file Table_1.DOCX]

| N=687 | 1 | 2 | 3 | 4 | 5 | 6 | 7 | 8 | 9 | 10 | 11 | 12 |
| --- | --- | --- | --- | --- | --- | --- | --- | --- | --- | --- | --- | --- |
| 1.I explore new ideas in various ways. | 0.864 | 0.478 | 0.200 | 0.118 | 0.345 | 0.126 | 0.280 | 0.195 | 0.345 | 0.320 | 0.219 | 0.231 |
| 2.I like to challenge myself with new things. | 0.478 | 0.992 | 0.198 | 0.124 | 0.402 | 0.118 | 0.267 | 0.121 | 0.281 | 0.296 | 0.213 | 0.237 |
| 3.I will never be satisfied until I have acquired all necessary knowledge about an idea. | 0.200 | 0.198 | 1.148 | 0.317 | 0.06 | 0.318 | 0.229 | 0.280 | 0.313 | 0.243 | 0.426 | 0.405 |
| 4.When I can’t reach the solution, I am uneasy and eager to reach it. | 0.118 | 0.124 | 0.317 | 0.777 | 0.069 | 0.307 | 0.131 | 0.188 | 0.141 | 0.184 | 0.371 | 0.228 |
| 5.Wherever I go, I explore new things and new experiences. | 0.345 | 0.402 | 0.060 | 0.069 | 0.888 | 0.102 | 0.279 | 0.114 | 0.308 | 0.272 | 0.053 | 0.188 |
| 6.I will think through a problem, till a clear and definite answer emerges. | 0.126 | 0.118 | 0.318 | 0.307 | 0.102 | 0.867 | 0.147 | 0.271 | 0.194 | 0.198 | 0.393 | 0.241 |
| 7.I am curious about everything. | 0.280 | 0.267 | 0.229 | 0.131 | 0.279 | 0.147 | 0.964 | 0.238 | 0.435 | 0.312 | 0.264 | 0.217 |
| 8.When something unexpected has happened, I will check until I find out the cause. | 0.195 | 0.121 | 0.280 | 0.188 | 0.114 | 0.271 | 0.238 | 0.909 | 0.300 | 0.214 | 0.29 | 0.188 |
| 9.I am very curious about the things that nobody has tried yet. | 0.345 | 0.281 | 0.313 | 0.141 | 0.308 | 0.194 | 0.435 | 0.300 | 1.217 | 0.547 | 0.352 | 0.297 |
| 10.I am willing to work on a task that nobody has tried yet. | 0.320 | 0.296 | 0.243 | 0.184 | 0.272 | 0.198 | 0.312 | 0.214 | 0.547 | 1.041 | 0.419 | 0.275 |
| 11.I will think many hours to solve a problem. | 0.219 | 0.213 | 0.426 | 0.371 | 0.053 | 0.393 | 0.264 | 0.290 | 0.352 | 0.419 | 1.322 | 0.412 |
| 12.When I learn things, I want to inquire thoroughly. | 0.231 | 0.237 | 0.405 | 0.228 | 0.188 | 0.241 | 0.217 | 0.188 | 0.297 | 0.275 | 0.412 | 0.865 |

Table S1. Inter-item Correlation Matrix for the 12-item English Version of the J-EC Scale
