# Supplementary material for: A Japan-origin motivational framework for diversive and specific curiosity: development of the English version of the Japanese Epistemic Curiosity scale
Source: Front Psychol. 2026 Apr 20;17:1762069. doi: 10.3389/fpsyg.2026.1762069 (PMC13136233; doi:10.3389/fpsyg.2026.1762069)
Supplement: Supplementary file 2 [file Table_2.DOCX]

|  | N=687 | 1 |  | 2 |  | 3 |  | 4 |  | 5 |  |
| --- | --- | --- | --- | --- | --- | --- | --- | --- | --- | --- | --- |
| 1 | Diversive Curiosity |  |  |  |  |  |  |  |  |  |  |
| 2 | Specific Curiosity | .45 | ^***^ |  |  |  |  |  |  |  |  |
| 3 | Big Five: Neuroticism | -.03 |  | .08 |  |  |  |  |  |  |  |
| 4 | Big Five: Openness | .34 | ^***^ | .25 | ^***^ | .03 |  |  |  |  |  |
| 5 | Big Five: Extroversion | .22 | ^***^ | -.10 | ^**^ | -.11 | ^**^ | .07 |  |  |  |
| 6 | Big Five: Agreeableness | .16 | ^***^ | .11 | ^**^ | .10 | ^**^ | .16 | ^***^ | .25 | ^***^ |
| 7 | Big Five: Conscientiousness | .00 |  | .19 | ^***^ | -.12 | ^**^ | -.10 | ^**^ | -.07 |  |
| 8 | Total-CEI-Ⅱ | .68 | ^***^ | .28 | ^***^ | -.17 | ^***^ | .26 | ^***^ | .34 | ^***^ |
| 9 | CEI-II: Embracing | .55 | ^***^ | .06 |  | -.20 | ^***^ | .18 | ^***^ | .43 | ^***^ |
| 10 | CEI-II: Stretching | .65 | ^***^ | .47 | ^***^ | -.09 | ^*^ | .29 | ^***^ | .16 | ^***^ |
| 11 | Need for Cognition Scale | .52 | ^***^ | .54 | ^***^ | .05 |  | .40 | ^***^ | -.01 |  |
| 12 | Need for Cognitive Closure scale: Ambiguity | -.08 | ^*^ | .23 | ^***^ | .29 | ^***^ | .01 |  | -.19 | ^***^ |
| 13 | Need for Cognitive Closure scale: Closed Mindedness | -.28 | ^***^ | -.17 | ^***^ | .07 |  | -.28 | ^***^ | -.05 |  |
| 14 | Need for Cognitive Closure scale: Decisiveness | -.01 |  | -.21 | ^***^ | -.21 | ^***^ | .01 |  | .01 |  |
| 15 | Need for Cognitive Closure scale: Preference to Order | -.15 | ^***^ | .18 | ^***^ | .11 | ^**^ | -.17 | ^***^ | -.16 | ^***^ |
| 16 | Need for Cognitive Closure scale: Predictability | -.26 | ^***^ | .11 | ^**^ | .22 | ^***^ | -.10 | ^**^ | -.26 | ^***^ |
| 17 | BIS/BAS scale: BAS Drive | .38 | ^***^ | .28 | ^***^ | -.05 |  | .09 | ^*^ | .26 | ^***^ |
| 18 | BIS/BAS scale: BAS Fun seeking | .42 | ^***^ | -.05 |  | -.08 | ^*^ | .21 | ^***^ | .41 | ^***^ |
| 19 | BIS/BAS scale: BAS Reward | .21 | ^***^ | .17 | ^***^ | .02 |  | .14 | ^***^ | .21 | ^***^ |
| 20 | BIS/BAS scale: BIS | -.11 | ^**^ | .14 | ^***^ | .52 | ^***^ | .05 |  | -.19 | ^***^ |

Table S2. Correlation Matrix and Descriptive Statistics for All Study Variables

|  | N=687 | 6 |  | 7 |  | 8 |  | 9 |  | 10 |  |
| --- | --- | --- | --- | --- | --- | --- | --- | --- | --- | --- | --- |
| 1 | Diversive Curiosity |  |  |  |  |  |  |  |  |  |  |
| 2 | Specific Curiosity |  |  |  |  |  |  |  |  |  |  |
| 3 | Big Five: Neuroticism |  |  |  |  |  |  |  |  |  |  |
| 4 | Big Five: Openness |  |  |  |  |  |  |  |  |  |  |
| 5 | Big Five: Extroversion |  |  |  |  |  |  |  |  |  |  |
| 6 | Big Five: Agreeableness |  |  |  |  |  |  |  |  |  |  |
| 7 | Big Five: Conscientiousness | .04 |  |  |  |  |  |  |  |  |  |
| 8 | Total-CEI-Ⅱ | .12 | ^**^ | -.05 |  |  |  |  |  |  |  |
| 9 | CEI-II: Embracing | .09 | ^*^ | -.14 | ^***^ | .90 | ^***^ |  |  |  |  |
| 10 | CEI-II: Stretching | .13 | ^***^ | .07 |  | .87 | ^***^ | .57 | ^***^ |  |  |
| 11 | Need for Cognition Scale | .15 | ^***^ | .06 |  | .44 | ^***^ | .23 | ^***^ | .57 | ^***^ |
| 12 | Need for Cognitive Closure scale: Ambiguity | .04 |  | .09 | ^*^ | -.26 | ^***^ | -.32 | ^***^ | -.12 | ^***^ |
| 13 | Need for Cognitive Closure scale: Closed Mindedness | -.12 | ^**^ | .07 | ^*^ | -.28 | ^***^ | -.22 | ^***^ | -.28 | ^***^ |
| 14 | Need for Cognitive Closure scale: Decisiveness | -.10 | ^**^ | -.12 | ^**^ | .08 | ^*^ | .12 | ^**^ | .02 |  |
| 15 | Need for Cognitive Closure scale: Preference to Order | .01 |  | .44 | ^***^ | -.25 | ^***^ | -.34 | ^***^ | -.08 | ^*^ |
| 16 | Need for Cognitive Closure scale: Predictability | -.04 |  | .21 | ^***^ | -.42 | ^***^ | -.50 | ^***^ | -.23 | ^***^ |
| 17 | BIS/BAS scale: BAS Drive | .11 | ^**^ | .05 |  | .39 | ^***^ | .31 | ^***^ | .39 | ^***^ |
| 18 | BIS/BAS scale: BAS Fun seeking | .18 | ^***^ | -.21 | ^***^ | .53 | ^***^ | .59 | ^***^ | .33 | ^***^ |
| 19 | BIS/BAS scale: BAS Reward | .28 | ^***^ | .05 |  | .20 | ^***^ | .17 | ^***^ | .19 | ^***^ |
| 20 | BIS/BAS scale: BIS | .21 | ^***^ | -.04 |  | -.31 | ^***^ | -.36 | ^***^ | -.17 | ^***^ |

|  | N=687 | 11 |  | 12 |  | 13 |  | 14 |  | 15 |  |
| --- | --- | --- | --- | --- | --- | --- | --- | --- | --- | --- | --- |
| 1 | Diversive Curiosity |  |  |  |  |  |  |  |  |  |  |
| 2 | Specific Curiosity |  |  |  |  |  |  |  |  |  |  |
| 3 | Big Five: Neuroticism |  |  |  |  |  |  |  |  |  |  |
| 4 | Big Five: Openness |  |  |  |  |  |  |  |  |  |  |
| 5 | Big Five: Extroversion |  |  |  |  |  |  |  |  |  |  |
| 6 | Big Five: Agreeableness |  |  |  |  |  |  |  |  |  |  |
| 7 | Big Five: Conscientiousness |  |  |  |  |  |  |  |  |  |  |
| 8 | Total-CEI-Ⅱ |  |  |  |  |  |  |  |  |  |  |
| 9 | CEI-II: Embracing |  |  |  |  |  |  |  |  |  |  |
| 10 | CEI-II: Stretching |  |  |  |  |  |  |  |  |  |  |
| 11 | Need for Cognition Scale |  |  |  |  |  |  |  |  |  |  |
| 12 | Need for Cognitive Closure scale: Ambiguity | -.14 | ^***^ |  |  |  |  |  |  |  |  |
| 13 | Need for Cognitive Closure scale: Closed Mindedness | -.39 | ^***^ | .30 | ^***^ |  |  |  |  |  |  |
| 14 | Need for Cognitive Closure scale: Decisiveness | .13 | ^***^ | -.44 | ^***^ | -.24 | ^***^ |  |  |  |  |
| 15 | Need for Cognitive Closure scale: Preference to Order | -.11 | ^**^ | .36 | ^***^ | .21 | ^***^ | -.32 | ^***^ |  |  |
| 16 | Need for Cognitive Closure scale: Predictability | -.18 | ^***^ | .61 | ^***^ | .38 | ^***^ | -.32 | ^***^ | .48 | ^***^ |
| 17 | BIS/BAS scale: BAS Drive | .20 | ^***^ | .01 |  | -.04 |  | -.16 | ^***^ | -.02 |  |
| 18 | BIS/BAS scale: BAS Fun seeking | .09 | ^*^ | -.18 | ^***^ | -.15 | ^***^ | -.01 |  | -.33 | ^***^ |
| 19 | BIS/BAS scale: BAS Reward | .04 |  | .13 | ^***^ | -.02 |  | -.28 | ^***^ | .07 |  |
| 20 | BIS/BAS scale: BIS | -.06 |  | .47 | ^***^ | .13 | ^***^ | -.31 | ^***^ | .26 | ^***^ |

|  | N=687 | 16 |  | 17 |  | 18 |  | 19 |  | 20 | M | SD | α |
| --- | --- | --- | --- | --- | --- | --- | --- | --- | --- | --- | --- | --- | --- |
| 1 | Diversive Curiosity |  |  |  |  |  |  |  |  |  | 3.51 | .67 | .76 |
| 2 | Specific Curiosity |  |  |  |  |  |  |  |  |  | 3.54 | .65 | .73 |
| 3 | Big Five: Neuroticism |  |  |  |  |  |  |  |  |  | 3.41 | .80 | .66 |
| 4 | Big Five: Openness |  |  |  |  |  |  |  |  |  | 3.75 | .69 | .69 |
| 5 | Big Five: Extroversion |  |  |  |  |  |  |  |  |  | 3.15 | .95 | .84 |
| 6 | Big Five: Agreeableness |  |  |  |  |  |  |  |  |  | 4.20 | .66 | .72 |
| 7 | Big Five: Conscientiousness |  |  |  |  |  |  |  |  |  | 3.30 | .85 | .73 |
| 8 | Total-CEI-Ⅱ |  |  |  |  |  |  |  |  |  | 3.05 | .71 | .84 |
| 9 | CEI-II: Embracing |  |  |  |  |  |  |  |  |  | 2.87 | .85 | .77 |
| 10 | CEI-II: Stretching |  |  |  |  |  |  |  |  |  | 3.24 | .75 | .77 |
| 11 | Need for Cognition Scale |  |  |  |  |  |  |  |  |  | 3.22 | .58 | .87 |
| 12 | Need for Cognitive Closure scale: Ambiguity |  |  |  |  |  |  |  |  |  | 4.11 | .88 | .51 |
| 13 | Need for Cognitive Closure scale: Closed Mindedness |  |  |  |  |  |  |  |  |  | 3.00 | .91 | .43 |
| 14 | Need for Cognitive Closure scale: Decisiveness |  |  |  |  |  |  |  |  |  | 2.80 | .82 | .53 |
| 15 | Need for Cognitive Closure scale: Preference to Order |  |  |  |  |  |  |  |  |  | 4.07 | 1.15 | .89 |
| 16 | Need for Cognitive Closure scale: Predictability |  |  |  |  |  |  |  |  |  | 3.69 | .98 | .67 |
| 17 | BIS/BAS scale: BAS Drive | -.12 | ^**^ |  |  |  |  |  |  |  | 2.65 | .53 | .78 |
| 18 | BIS/BAS scale: BAS Fun seeking | -.41 | ^***^ | .34 | ^***^ |  |  |  |  |  | 3.00 | .57 | .74 |
| 19 | BIS/BAS scale: BAS Reward | -.02 |  | .41 | ^***^ | .40 | ^***^ |  |  |  | 3.31 | .43 | .70 |
| 20 | BIS/BAS scale: BIS | .41 | ^***^ | -.10 | ^**^ | -.13 | ^***^ | .18 | ^***^ |  | 3.28 | .50 | .81 |
